# 3-Acet­oxy-2-naphthoic acid

**DOI:** 10.1107/S1600536810040365

**Published:** 2010-10-20

**Authors:** Bruno S. Souza, Ramon Vitto, Faruk Nome, Anthony J. Kirby, Adailton J. Bortoluzzi

**Affiliations:** a Depto. de Química, Universidade Federal de Santa Catarina, 88040-900 Florianópolis, Santa Catarina, Brazil; b University Chemical Laboratory, Cambridge University, Cambridge CB2 1EW, England

## Abstract

In the title compound, C_13_H_10_O_4_, an analog of acetyl­salicylic acid, the naphthalene unit is twisted slightly due to *ortho* disubstitution [dihedral angle between conjugated rings system in the naphthalene unit = 2.0 (2)°]. The mean planes of the carb­oxy­lic and ester groups are almost coplanar and perpendicular, respectively, to the mean plane of the conjugated aromatic system, making dihedral angles of 8.9 (3) and 89.3 (1)°. In the crystal, mol­ecules are paired through their carb­oxy­lic groups by the typical centrosymmetric O—H⋯O inter­actions with *R*_2_^2^(8) hydrogen-bond motifs. In addition, several weak C—H⋯O inter­molecular contacts are also observed. Finally, the mol­ecules are stacked along crystallographic [100] and [010] directions.

## Related literature

This work was undertaken as part of our study on the relationship between conformation and reactivity in the hydrolysis reactions of esters bearing neighboring catalytic groups. For the synthesis, see: Bergeron *et al.* (1996 ▶). For related structures, see: Souza *et al.* (2007 ▶); Gu *et al.* (2001 ▶); Wilson (2002) ▶. Besides electronic effects, intra­molecular reactions depend on the spatial relationship of the reacting groups, see: Orth *et al.* (2010 ▶).
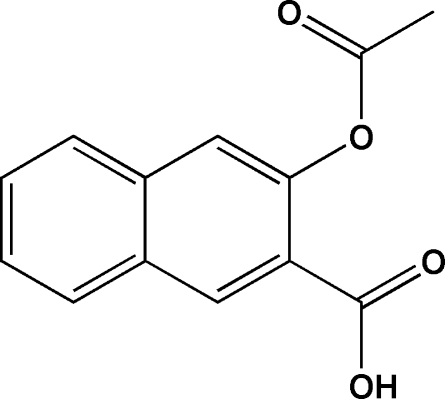


## Experimental

### 

#### Crystal data

C_13_H_10_O_4_
*M* *_r_* = 230.21
Monoclinic, 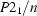

*a* = 10.235 (2) Å
*b* = 4.739 (2) Å
*c* = 23.0873 (16) Å
β = 101.060 (11)°
*V* = 1099.0 (5) Å^3^
*Z* = 4
Mo *K*α radiation
μ = 0.10 mm^−1^
*T* = 295 K
0.50 × 0.33 × 0.10 mm

#### Data collection

Enraf–Nonius CAD-4 diffractometer
1999 measured reflections
1950 independent reflections
1214 reflections with *I* > 2σ(*I*)
*R*_int_ = 0.026
3 standard reflections every 200 reflections  intensity decay: 1%

#### Refinement

*R*[*F* ^2^ > 2σ(*F* ^2^)] = 0.063
*wR*(*F* ^2^) = 0.204
*S* = 1.05
1950 reflections
155 parameters
H-atom parameters constrained
Δρ_max_ = 0.26 e Å^−3^
Δρ_min_ = −0.26 e Å^−3^

### 

Data collection: *CAD-4 Software* (Enraf–Nonius, 1989 ▶); cell refinement: *SET4* in *CAD-4 Software*; data reduction: *HELENA* (Spek, 1996 ▶); program(s) used to solve structure: *SIR97* (Altomare *et al.*, 1999 ▶); program(s) used to refine structure: *SHELXL97* (Sheldrick, 2008 ▶); molecular graphics: *PLATON* (Spek, 2009 ▶); software used to prepare material for publication: *SHELXL97* ▶.

## Supplementary Material

Crystal structure: contains datablocks global, I. DOI: 10.1107/S1600536810040365/rk2238sup1.cif

Structure factors: contains datablocks I. DOI: 10.1107/S1600536810040365/rk2238Isup2.hkl

Additional supplementary materials:  crystallographic information; 3D view; checkCIF report

## Figures and Tables

**Table 1:** Hydrogen-bond geometry (Å, °)

*D*—H⋯*A*	*D*—H	H⋯*A*	*D*⋯*A*	*D*—H⋯*A*
O2—H2⋯O1^i^	0.91	1.74	2.636 (3)	171
C7—H7⋯O4^ii^	0.93	2.71	3.368 (5)	128
C13—H13*B*⋯O4^iii^	0.96	2.52	3.435 (5)	160

Symmetry codes: (i) 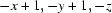; (ii) 
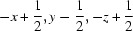
; (iii) 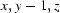.

**Fig. 1.**
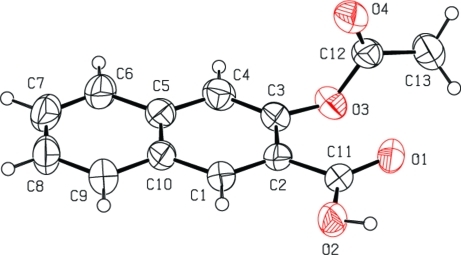
The molecular structure of the title compound with the atom labeling scheme. Displacement ellipsoids are shown at the 40% probability level. H atoms are presented as a small spheres of arbitrary radius.

**Fig. 2.**
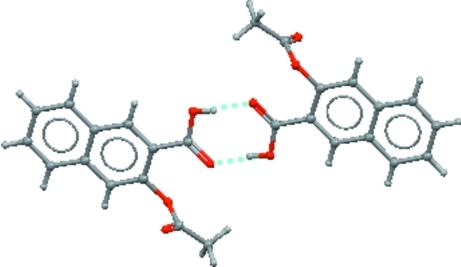
Molecules paired by centrosymmetric R22(8) hydrogen bonds.

**Fig. 3.**
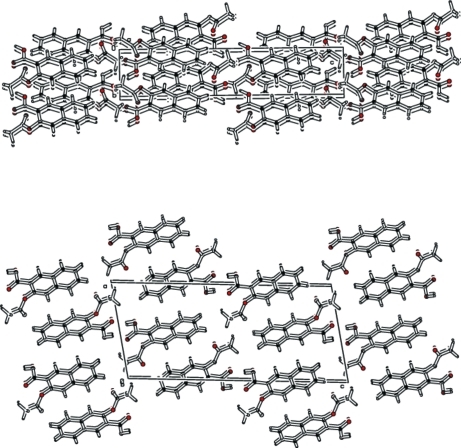
Molecules of I stacked along [100] direction (top) and along [010] direction (bottom).

## supplementary crystallographic information

### Comment

Besides electronic effects, intramolecular reactions depend on the spatial relationship of the reacting groups (Orth *et al.*, 2010). Subtle changes in the structure of similar compounds may lead to different relationships between nucleophilic-electrophilic centers within similar molecules, leading to different reaction mechanisms in solution. On this basis, we are currently studying the hydrolysis of a series of structurally related naphthyl esters. In a previous report (Souza *et al.*, 2007), we published the structure of 2-carboxy-1-naphthyl acetate, a naphthoic acid bearing an *ortho*-ester group. In that structure, the dihedral angle between the aromatic mean plane and the ester group is 80.34 (5)°, considerably smaller than the one observed in α-naphthyl acetate (86.50°) (Gu *et al.*, 2001), and we explained this difference in terms of an attractive interaction between the ester and the acid groups in 2-carboxy-1-naphthyl acetate. In the current work, we report the structure of 3-acetoxy-2-naphthoic acid I (Fig. 1), C_13_H_10_O_4_, an acetylsalicylic analog. In this system the dihedral angle between acetoxy and aromatic mean planes is 89.3 (1)°, while the carboxylic acid group and naphthalene ring are almost coplanar with dihedral angle of 8.9 (3)°. Packing is controlled by carboxylic acid dimer formation, involving centrosymmetric O2–H···O1 interactions (*R*^2^_2_(8) hydrogen bond pattern, Fig. 2). Several weak C–H···O intermolecular contacts are also observed. Finally, the molecules are stacked along crystallographic [100] and [010] directions (Fig. 3).

### Experimental

The title compound was prepared by following the procedure reported by Bergeron *et al.*, (1996). Concentrated sulfuric acid (10 drops) was added to a refluxing mixture of 3-hydroxy-2-naphthoic acid (3.50 g, 18.6 mmol) in acetic anhydride (8 ml, 89.7 mmol). The mixture was kept under reflux for 10 additional minutes and, after cooling to room temperature, the pale solid was filtered off and recrystallized in aqueous ethanol. The 10 mg of the prepared 3-acetoxy-2-naphthoic acid were dissolved in 5 ml of dry CHCl_3_ in a 10 ml glass vial and the flask was kept in a saturated petroleum ether (313-333 K) atmosphere at 293 K, giving the title compound as pale yellow crystals that melt at 458-459 K.

### Refinement

All non-H atoms were refined with anisotropic displacement parameters. H atoms were placed at their idealized positions with distances of 0.93Å for C–H_Ar_ and 0.96Å for CH_3_ group. Their *U*_iso_H were fixed at 1.2 and 1.5 times *U*_eq_C of the preceding atom for aromatic and methyl group, respectively. The hydrogen atom of the acid group was located from the Fourier difference map and treated using a riding model with *U*_iso_H = 1.2*U*_eq_O.

### Crystal data

**Table:**

C_13_H_10_O_4_	*F*(000) = 480
*M**_r_* = 230.21	*D*_x_ = 1.391 Mg m^−^^3^
Monoclinic, *P*2_1_/*n*	Mo *K*α radiation, λ = 0.71073 Å
Hall symbol: -P 2yn	Cell parameters from 25 reflections
*a* = 10.235 (2) Å	θ = 6.5–18.5°
*b* = 4.739 (2) Å	µ = 0.10 mm^−^^1^
*c* = 23.0873 (16) Å	*T* = 295 K
β = 101.060 (11)°	Block, pale yellow
*V* = 1099.0 (5) Å^3^	0.50 × 0.33 × 0.10 mm
*Z* = 4	

### Data collection

**Table:**

Enraf–Nonius CAD-4 diffractometer	*R*_int_ = 0.026
Radiation source: fine-focus sealed tube	θ_max_ = 25.1°, θ_min_ = 1.8°
graphite	*h* = −11→12
ω–/2θ scans	*k* = −5→0
1999 measured reflections	*l* = −27→0
1950 independent reflections	3 standard reflections every 200 reflections
1214 reflections with *I* > 2σ(*I*)	intensity decay: 1%

### Refinement

**Table:**

Refinement on *F*^2^	Primary atom site location: structure-invariant direct methods
Least-squares matrix: full	Secondary atom site location: difference Fourier map
*R*[*F*^2^ > 2σ(*F*^2^)] = 0.063	Hydrogen site location: inferred from neighbouring sites
*wR*(*F*^2^) = 0.204	H-atom parameters constrained
*S* = 1.05	*w* = 1/[σ^2^(*F*_o_^2^) + (0.1252*P*)^2^ + 0.1035*P*] where *P* = (*F*_o_^2^ + 2*F*_c_^2^)/3
1950 reflections	(Δ/σ)_max_ < 0.001
155 parameters	Δρ_max_ = 0.26 e Å^−^^3^
0 restraints	Δρ_min_ = −0.26 e Å^−^^3^

### Fractional atomic coordinates and isotropic or equivalent isotropic displacement parameters (Å^2^)

**Table:**

	*x*	*y*	*z*	*U*_iso_*/*U*_eq_	
C1	0.5573 (3)	0.3368 (7)	0.18259 (12)	0.0566 (8)	
H1	0.6184	0.4763	0.1779	0.068*	
C2	0.4810 (3)	0.2182 (6)	0.13351 (12)	0.0518 (7)	
C3	0.3930 (3)	−0.0003 (6)	0.14168 (12)	0.0540 (8)	
C4	0.3780 (3)	−0.0817 (7)	0.19648 (14)	0.0632 (9)	
H4	0.3170	−0.2222	0.2006	0.076*	
C5	0.4544 (3)	0.0454 (7)	0.24728 (13)	0.0599 (8)	
C6	0.4428 (3)	−0.0331 (9)	0.30541 (14)	0.0737 (10)	
H6	0.3804	−0.1680	0.3110	0.088*	
C7	0.5213 (4)	0.0861 (9)	0.35239 (15)	0.0806 (11)	
H7	0.5127	0.0308	0.3901	0.097*	
C8	0.6136 (4)	0.2872 (9)	0.34600 (14)	0.0815 (11)	
H8	0.6669	0.3659	0.3792	0.098*	
C9	0.6278 (3)	0.3735 (8)	0.29060 (13)	0.0736 (10)	
H9	0.6906	0.5097	0.2863	0.088*	
C10	0.5463 (3)	0.2538 (7)	0.24028 (12)	0.0564 (8)	
C11	0.4918 (3)	0.3266 (7)	0.07423 (12)	0.0543 (7)	
C12	0.2108 (3)	−0.0557 (7)	0.06310 (13)	0.0583 (8)	
C13	0.1651 (3)	−0.2253 (8)	0.00866 (15)	0.0770 (10)	
H13A	0.0772	−0.1662	−0.0098	0.115*	
H13B	0.1635	−0.4215	0.0188	0.115*	
H13C	0.2250	−0.1974	−0.0181	0.115*	
O1	0.4153 (2)	0.2552 (5)	0.02898 (9)	0.0680 (7)	
O2	0.5866 (2)	0.5076 (6)	0.07447 (9)	0.0768 (8)	
H2	0.5764	0.5907	0.0385	0.092*	
O3	0.32733 (19)	−0.1555 (4)	0.09356 (9)	0.0623 (6)	
O4	0.1549 (2)	0.1389 (6)	0.07911 (10)	0.0802 (8)	

### Atomic displacement parameters (Å^2^)

**Table:**

	*U*^11^	*U*^22^	*U*^33^	*U*^12^	*U*^13^	*U*^23^
C1	0.0497 (15)	0.073 (2)	0.0460 (16)	0.0003 (15)	0.0077 (12)	0.0016 (14)
C2	0.0447 (14)	0.0656 (18)	0.0458 (15)	0.0087 (13)	0.0105 (12)	0.0041 (13)
C3	0.0466 (15)	0.0625 (18)	0.0508 (16)	0.0069 (14)	0.0043 (12)	0.0017 (14)
C4	0.0547 (17)	0.071 (2)	0.0629 (19)	−0.0013 (15)	0.0080 (14)	0.0167 (16)
C5	0.0536 (17)	0.073 (2)	0.0530 (17)	0.0156 (16)	0.0112 (13)	0.0124 (15)
C6	0.073 (2)	0.096 (3)	0.0540 (19)	0.0135 (19)	0.0171 (16)	0.0212 (18)
C7	0.089 (2)	0.105 (3)	0.050 (2)	0.029 (2)	0.0195 (18)	0.0172 (19)
C8	0.089 (2)	0.109 (3)	0.0429 (18)	0.021 (2)	0.0032 (16)	−0.0017 (18)
C9	0.071 (2)	0.097 (3)	0.0500 (18)	0.0015 (19)	0.0058 (15)	−0.0016 (17)
C10	0.0523 (16)	0.070 (2)	0.0465 (16)	0.0156 (15)	0.0082 (13)	0.0037 (14)
C11	0.0464 (15)	0.0720 (19)	0.0457 (16)	0.0021 (15)	0.0116 (12)	−0.0024 (14)
C12	0.0507 (16)	0.067 (2)	0.0567 (17)	0.0070 (15)	0.0100 (13)	0.0112 (16)
C13	0.069 (2)	0.092 (3)	0.064 (2)	0.0046 (19)	−0.0036 (15)	−0.0056 (18)
O1	0.0652 (13)	0.0970 (16)	0.0415 (11)	−0.0110 (12)	0.0096 (9)	−0.0044 (11)
O2	0.0623 (13)	0.120 (2)	0.0481 (12)	−0.0266 (14)	0.0093 (9)	0.0100 (12)
O3	0.0549 (12)	0.0653 (13)	0.0612 (13)	0.0042 (10)	−0.0029 (9)	0.0011 (10)
O4	0.0618 (13)	0.1063 (19)	0.0698 (15)	0.0223 (14)	0.0057 (11)	−0.0064 (13)

### Geometric parameters (Å, °)

**Table:**

C1—C2	1.368 (4)	C7—H7	0.9300
C1—C10	1.414 (4)	C8—C9	1.377 (5)
C1—H1	0.9300	C8—H8	0.9300
C2—C3	1.409 (4)	C9—C10	1.412 (4)
C2—C11	1.486 (4)	C9—H9	0.9300
C3—C4	1.359 (4)	C11—O1	1.227 (3)
C3—O3	1.393 (3)	C11—O2	1.295 (3)
C4—C5	1.413 (4)	C12—O4	1.181 (4)
C4—H4	0.9300	C12—O3	1.349 (3)
C5—C10	1.395 (4)	C12—C13	1.490 (4)
C5—C6	1.419 (4)	C13—H13A	0.9600
C6—C7	1.344 (5)	C13—H13B	0.9600
C6—H6	0.9300	C13—H13C	0.9600
C7—C8	1.370 (5)	O2—H2	0.9071
			
C2—C1—C10	122.0 (3)	C7—C8—H8	119.9
C2—C1—H1	119.0	C9—C8—H8	119.9
C10—C1—H1	119.0	C8—C9—C10	119.6 (4)
C1—C2—C3	118.0 (3)	C8—C9—H9	120.2
C1—C2—C11	119.3 (3)	C10—C9—H9	120.2
C3—C2—C11	122.7 (3)	C5—C10—C9	119.6 (3)
C4—C3—O3	118.0 (3)	C5—C10—C1	118.9 (3)
C4—C3—C2	121.5 (3)	C9—C10—C1	121.5 (3)
O3—C3—C2	120.3 (2)	O1—C11—O2	122.8 (3)
C3—C4—C5	120.6 (3)	O1—C11—C2	122.8 (3)
C3—C4—H4	119.7	O2—C11—C2	114.4 (2)
C5—C4—H4	119.7	O4—C12—O3	122.9 (3)
C10—C5—C4	118.9 (3)	O4—C12—C13	126.3 (3)
C10—C5—C6	118.4 (3)	O3—C12—C13	110.7 (3)
C4—C5—C6	122.7 (3)	C12—C13—H13A	109.5
C7—C6—C5	120.5 (4)	C12—C13—H13B	109.5
C7—C6—H6	119.8	H13A—C13—H13B	109.5
C5—C6—H6	119.8	C12—C13—H13C	109.5
C6—C7—C8	121.6 (3)	H13A—C13—H13C	109.5
C6—C7—H7	119.2	H13B—C13—H13C	109.5
C8—C7—H7	119.2	C11—O2—H2	109.2
C7—C8—C9	120.3 (3)	C12—O3—C3	118.3 (2)
			
C10—C1—C2—C3	−2.4 (4)	C6—C5—C10—C9	2.1 (4)
C10—C1—C2—C11	176.4 (3)	C4—C5—C10—C1	1.8 (4)
C1—C2—C3—C4	3.6 (4)	C6—C5—C10—C1	−179.1 (3)
C11—C2—C3—C4	−175.2 (3)	C8—C9—C10—C5	−1.3 (5)
C1—C2—C3—O3	−170.7 (2)	C8—C9—C10—C1	179.8 (3)
C11—C2—C3—O3	10.5 (4)	C2—C1—C10—C5	−0.3 (4)
O3—C3—C4—C5	172.3 (2)	C2—C1—C10—C9	178.6 (3)
C2—C3—C4—C5	−2.1 (5)	C1—C2—C11—O1	−170.7 (3)
C3—C4—C5—C10	−0.6 (5)	C3—C2—C11—O1	8.0 (5)
C3—C4—C5—C6	−179.7 (3)	C1—C2—C11—O2	7.7 (4)
C10—C5—C6—C7	−1.7 (5)	C3—C2—C11—O2	−173.5 (2)
C4—C5—C6—C7	177.4 (3)	O4—C12—O3—C3	−8.7 (4)
C5—C6—C7—C8	0.5 (6)	C13—C12—O3—C3	171.9 (3)
C6—C7—C8—C9	0.3 (6)	C4—C3—O3—C12	98.6 (3)
C7—C8—C9—C10	0.1 (5)	C2—C3—O3—C12	−86.8 (3)
C4—C5—C10—C9	−177.1 (3)		

### Hydrogen-bond geometry (Å, °)

**Table:**

*D*—H···*A*	*D*—H	H···*A*	*D*···*A*	*D*—H···*A*
O2—H2···O1^i^	0.91	1.74	2.636 (3)	171
C7—H7···O4^ii^	0.93	2.71	3.368 (5)	128
C13—H13B···O4^iii^	0.96	2.52	3.435 (5)	160

Symmetry codes: (i) −*x*+1, −*y*+1, −*z*; (ii) −*x*+1/2, *y*−1/2, −*z*+1/2; (iii) *x*, *y*−1, *z*.

## References

[bb1] Altomare, A., Burla, M. C., Camalli, M., Cascarano, G. L., Giacovazzo, C., Guagliardi, A., Moliterni, A. G. G., Polidori, G. & Spagna, R. (1999). *J. Appl. Cryst.* **32**, 115–119.

[bb2] Bergeron, R. J., Wiegand, J., Wollenweber, M., McManis, J. S., Algee, S. E. & Ratliff-Thompson, K. (1996). *J. Med. Chem.* **39**, 1575–1581. 10.1021/jm9508752 8648596

[bb3] Enraf–Nonius (1989). *CAD-4 Software* Enraf–Nonius, Delft, The Netherlands.

[bb4] Gu, W., Abdallah, D. J. & Weiss, R. G. (2001). *Photochem. Photobiol. A*, **139**, 79–87.

[bb5] Orth, E. S., Brandão, T. A. S., Souza, B. S., Pliego, J. R., Vaz, B. G., Eberlin, M. N., Kirby, A. J. & Nome, F. (2010). *J. Am. Chem. Soc.* **132**, 8513–8523. 10.1021/ja1034733 20509675

[bb6] Sheldrick, G. M. (2008). *Acta Cryst.* A**64**, 112–122. 10.1107/S0108767307043930 18156677

[bb7] Souza, B. S., Bortoluzzi, A. J. & Nome, F. (2007). *Acta Cryst.* E**63**, o4523.

[bb8] Spek, A. L. (1996). *HELENA* University of Utrecht, The Netherlands.

[bb9] Spek, A. L. (2009). *Acta Cryst.* D**65**, 148–155. 10.1107/S090744490804362X PMC2631630 19171970

[bb10] Wilson, C. C. (2002). *New J. Chem.* **26**, 1733–1739.

